# The Long Non-Coding RNA *RHPN1-AS1* Promotes Uveal Melanoma Progression

**DOI:** 10.3390/ijms18010226

**Published:** 2017-01-23

**Authors:** Linna Lu, Xiaoyu Yu, Leilei Zhang, Xia Ding, Hui Pan, Xuyang Wen, Shiqiong Xu, Yue Xing, Jiayan Fan, Shengfang Ge, He Zhang, Renbing Jia, Xianqun Fan

**Affiliations:** Department of Ophthalmology, Ninth People’s Hospital, Shanghai Jiao Tong University School of Medicine, Shanghai 200025, China; drlulinna@126.com (L.L.); yuxiaoyunzb@163.com (X.Y.); a789leilei@126.com (L.Z.); abcdingxia@126.com (X.D.); xypanhui@163.com (H.P.); winston-a@hotmail.com (X.W.); 15721201988@163.com (S.X.); fishashorejessica@163.com (Y.X.); fanjiayan1118@126.com (J.F.); geshengfang@sjtu.edu.cn (S.G.); zhanghe@sjtu.edu.cn (H.Z.)

**Keywords:** lncRNA, *RHPN1-AS1*, uveal melanoma, migration

## Abstract

Increasing evidence suggests that aberrant long non-coding RNAs (lncRNAs) are significantly correlated with the pathogenesis, development and metastasis of cancers. *RHPN1 antisense RNA 1* (*RHPN1-AS1*) is a 2030-bp transcript originating from human chromosome 8q24. However, the role of *RHPN1-AS1* in uveal melanoma (UM) remains to be clarified. In this study, we aimed to elucidate the molecular function of *RHPN1-AS1* in UM. The RNA levels of *RHPN1-AS1* in UM cell lines were examined using the quantitative real-time polymerase chain reaction (qRT-PCR). Short interfering RNAs (siRNAs) were designed to quench *RHPN1-AS1* expression, and UM cells stably expressing short hairpin (sh) *RHPN1-AS1* were established. Next, the cell proliferation and migration abilities were determined using a colony formation assay and a transwell migration/invasion assay. A tumor xenograft model in nude mice was established to confirm the function of *RHPN1-AS1* in vivo. *RHPN1-AS1* was significantly upregulated in a number of UM cell lines compared with the normal human retinal pigment epithelium (RPE) cell line. *RHPN1-AS1* knockdown significantly inhibited UM cell proliferation and migration in vitro and in vivo. Our data suggest that *RHPN1-AS1* could be an oncoRNA in UM, which may serve as a candidate prognostic biomarker and target for new therapies in malignant UM.

## 1. Introduction

Uveal melanoma (UM) is the most common primary intraocular tumor in adults, which arises from uveal melanocytes and has a strong propensity to metastasize [1]. The most frequent metastatic site is the liver, followed by the lung and soft tissues [2]. Although optimal treatments (surgery or radiation) have been developed for primary tumors, there are no effective therapies for metastatic UM. In the Collaborative Ocular Melanoma Study, the prognosis for metastatic UM was found to be poor, with a one-year overall mortality rate of 80%–87% [3,4]. Highly metastatic UM tumors are usually caused by the loss of one copy of chromosome 3 and the gain of an additional 8q [5]. Recent studies have shown that somatic mutations occur in a mutually exclusive pattern in Guanine Nucleotide-Binding Protein α-Q (*GNAQ*) or Guanine Nucleotide-Binding Protein G α-11 (*GNA11*) in ~83% of UM cases [6], and inactivating somatic mutations of BRCA associated protein-1 (*BAP1*) occur in ~84% of metastasizing tumors [7].

Research on mammalian transcriptomes suggests that only 1.5% of the human genome encodes protein-coding genes [8]. However, recent data from the Encyclopedia of DNA Elements (ENCODE) consortium indicate that around 70% of human genome is actively transcribed, generating a vast range of non-coding RNAs (ncRNAs) [9]. Based on the transcript length, ncRNAs are classified into small ncRNAs (<200 nt) and long ncRNAs (lncRNAs, >200 nt). Although lncRNAs share common features with mRNAs, as many of them are transcribed by RNA polymerase II, spliced, and 5′-capped [9], lncRNAs also have several distinct features. Some lncRNAs are evolutionarily conserved, implying that they are functionally important [10]. Genome screening studies indicate that lncRNAs are often expressed in a tissue-, developmental stage- or disease-specific pattern [11]. In addition, there is evidence indicating that lncRNAs are important regulatory molecules at various levels, including involvement in chromatin modification, transcription, and post-transcriptional processing [8]. Previous studies have shown that the lncRNA *ROR* occupies and activates the tescalcin (TESC) promoter and promotes metastasis [12]. However, the function of lncRNAs in UM is not well understood.

Aberrant expression of lncRNAs has been shown to contribute to tumorigenesis in cancers such as prostate cancer, gastric cancer and leukemia [13,14,15], and we previously conducted a study of cDNA microarrays in UM samples and normal tissues (data unpublished). We found that RHPN1 Antisense RNA 1 (*RHPN1-AS1*) was highly expressed in UM cancerous tissues compared to normal tissues. Down regulating *RHPN1-AS1* in a variety of UM cells inhibitedcolony formation, migration and invasionin vitroand tumor growthin vivo. Our results show, for the first time, that *RHPN1-AS1* plays a potential role in the progression of UM. Thus, this lncRNA might be an attractive biomarker and therapeutic target in UM.

## 2. Results

### 2.1. RHPN1-AS1 Is a Cytoplasmic lncRNA That Is Upregulated in Uveal Melanoma (UM)

**Figure 1:**
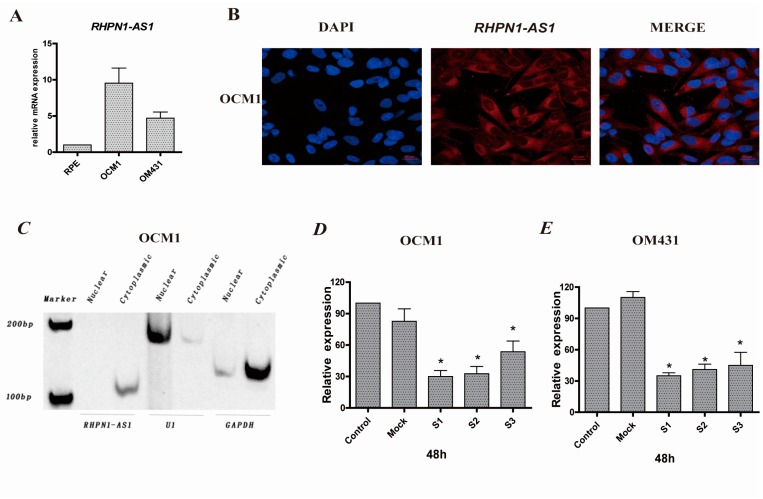
*RHPN1-AS1*, a cytoplasmic lncRNA, is aberrantly expressed in UM cell lines. (**A**) *RHPN1-AS1* expression was measured by real-time PCR in different UM cells and normal cell (RPE). *RHPN1-AS1* presented higher expression in melanoma cell lines OCM1 and OM431 than RPE cells; (**B**) *RHPN1-AS1* is cytoplasmically distributed. RNA fluorescence in situ hybridization (FISH) (red) was performed with cy3-labeled probes that recognizing *RHPN1-AS1*. The scale bars represent 20 µm; (**C**) *RHPN1-AS1* is associated with the cytoplasmic fractions. Total RNAs from OCM1 cells were separated into cytoplasmic and nuclear soluble fractions. *U1*, *GAPDH* were used as controls; (**D**,**E**) The interference rate was detected 48 h after *RHPN1-AS1* siRNAs transfection in OCM1 and OM431 cells. Triplicate assays were performed for each sample and the relative level of *RHPN1-AS1* was normalized to the *GAPDH*. (* *p* < 0.05).

To investigate the expression profiling and the role of lncRNAs in UM, microarrays containing probes targeting 12,784 lncRNAs were used in uveal melanoma samples and noncancerous tissues (unpublished data). We found that the expression level of *RHPN1-AS1* was upregulated in UM tissues compared with normal tissues. Furthermore, the expression level of *RHPN1-AS1* was detected by qRT-PCR in a variety of UM cell lines, including OCM1 and OM431. RPE cells served as controls. Compared with the normal RPE cells, *RHPN1-AS1* was significantly overexpressed in UM cells (Figure 1A).

The non-coding nature of *RHPN1-AS1* was confirmed by coding-potential analysis (Figure S1). Prediction of putative proteins encoded by *RHPN1-AS1* using Open Reading Frame Finder and the condon substitution frequency scores (CSF) of *RHPN1-AS1* indicated that *RHPN1-AS1* lacks protein coding potential. Next, to determine the subcellular localization of *RHPN1-AS1*, we performed RNA fluorescence in situ hybridization (FISH) analyses using cy3-labeled probes that recognize *RHPN1-AS1*. We found that fluorescent signals (red) appeared in cytoplasm (Figure 1B), suggesting that *RHPN1-AS1* is located in the cytoplasm. This was further confirmed by nuclear/cytoplasmic RNA fractionation, which revealed that *RHPN1-AS1* is associated with the cytoplasmic fractions (Figure 1C).

### 2.2. RHPN1-AS1 Knockdown Cell Lines

To decipher the potential role of *RHPN1-AS1* in UM, three siRNAs were designed to knockdown *RHPN1-AS1* expression. *RHPN1-AS1* expression was significantly knocked down after transfection with these siRNAs in OM431 and OCM1 cells. The highest interference rate was about 70% in OM431 cells and 60% in OCM1 cells (*p* < 0.05 vs. controls and Mock, Figure 1D,E). *RHPN1-AS1-si1* and *RHPN1-AS1-si2* were was selected for use in subsequent experiments.

**Figure 2:**
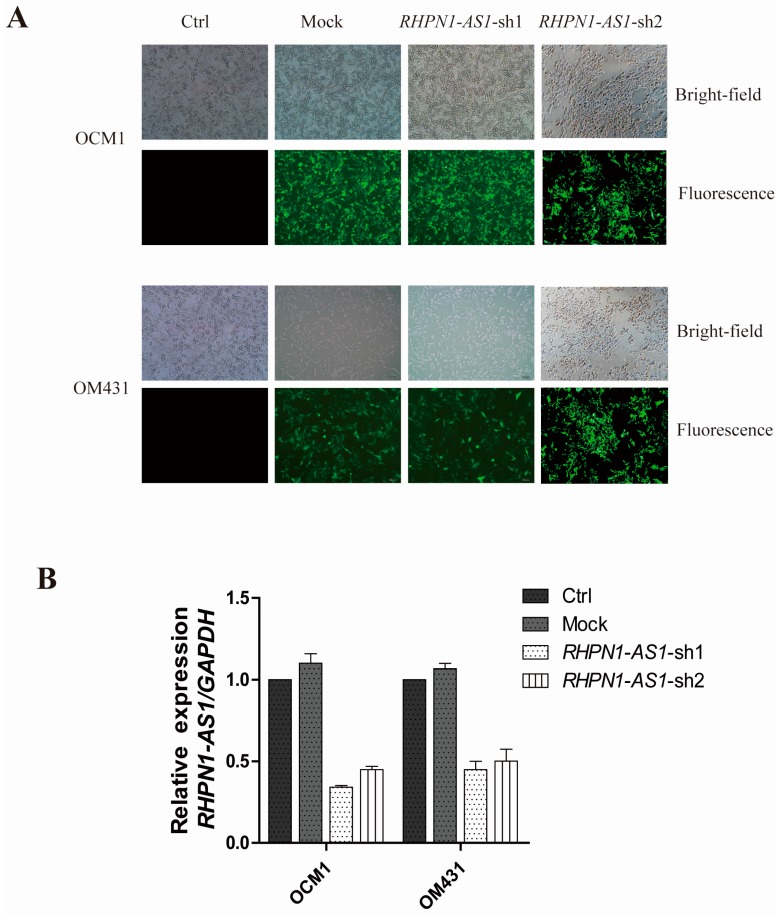
*RHPN1-AS1* knockdown by two shRNAs: (**A**) EGFP was used to track the expression of *RHPN1-AS1* shRNAs and control vectors in OCM1 and OM431 cells. The scale bars represent 100 μm; and (**B**) detection of *RHPN1-AS1* mRNA level in OCM1 and OM431 cells after shRNA-mediated knockdown of *RHPN1-AS1* by qRT-PCR.

The pGIPZ-shRNA vectors and empty pGIPZ control vectors with an enhanced green fluorescent protein (EGFP) maker was packaged into lenti-viruses and transfected human OCM1 and OM431 cells, named *RHPN1-AS1-sh1*, *RHPN1-AS1-sh2* and mock, respectively. Transfection efficiency was determined by GFP expression. GFP expression was seen in the mock, *RHPN1-AS1-sh1* and *-sh2* cells (Figure 2A). The knockdown efficiency was measured by qRT-PCR. *RHPN1-AS1* expression was significantly decreased in *RHPN1-AS1-sh1* and *RHPN1-AS1-sh2* transfected cells (Figure 2B).

### 2.3. Down-Regulation of RHPN1-AS1 Inhibited Cell Proliferation, Migrationand Invasion In Vitro

**Figure 3:**
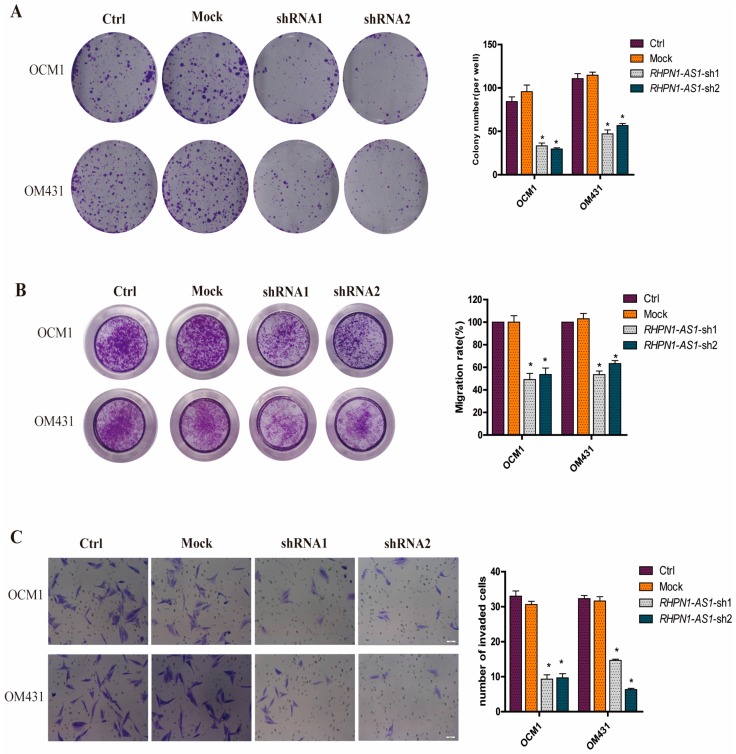
Knockdown of *RHPN1-AS1* inhibits proliferation, migration and invasion of UM cells in vitro. (**A**) Colony formation assays were performed to evaluate the effect of *RHPN1-AS1* on growth of UM cells after knockdown *RHPN1-AS1*. * *p* < 0.05; (**B**) The effect of *RHPN1-AS1* downregulation on the migration ability of OCM1 and OM431 cells was assessed using transwell assay. * *p* < 0.05; (**C**) Effects of *RHPN1-AS1* knockdown on the invasion potency of OCM1 and OM431 cells were detected using matrigel invasion assay. The scale bars represent 50 µm. * *p* < 0.05.

Next, we investigated whether the characteristics of the tumor cells were altered after *RHPN1-AS1* knockdown. We first examined the colony formation ability of *shRHPN1-AS1-*OCM1 cells and *shRHPN1-AS1-*OM431 cells compared to the control and mock groups using the colony formation assay. The number of colonies was significantly decreased after *RHPN1-AS1* knockdown (*p* < 0.05, Figure 3A). We also examined the effect of *RHPN1-AS1* knockdown on the migration and invasion ability of UM cells. The results demonstrated that the *RHPN1-AS1* knockdown inhibited UM cell migration by ~55% in OCM1 cells and by ~50% in OM431 cells, respectively (*p* < 0.05, Figure 3B) using *trans-*well assay. The matrigel invasion assay also showed that *RHPN1-AS1* knockdown in UM cells caused a significant decrease in cell invasion (*p* < 0.05, Figure 3C). These data indicate that *RHPN1-AS1* plays a regulatory role in tumor progression.

### 2.4. Down-Regulation of RHPN1-AS1 Decreased Xenograft Growth In Vivo

**Figure 4:**
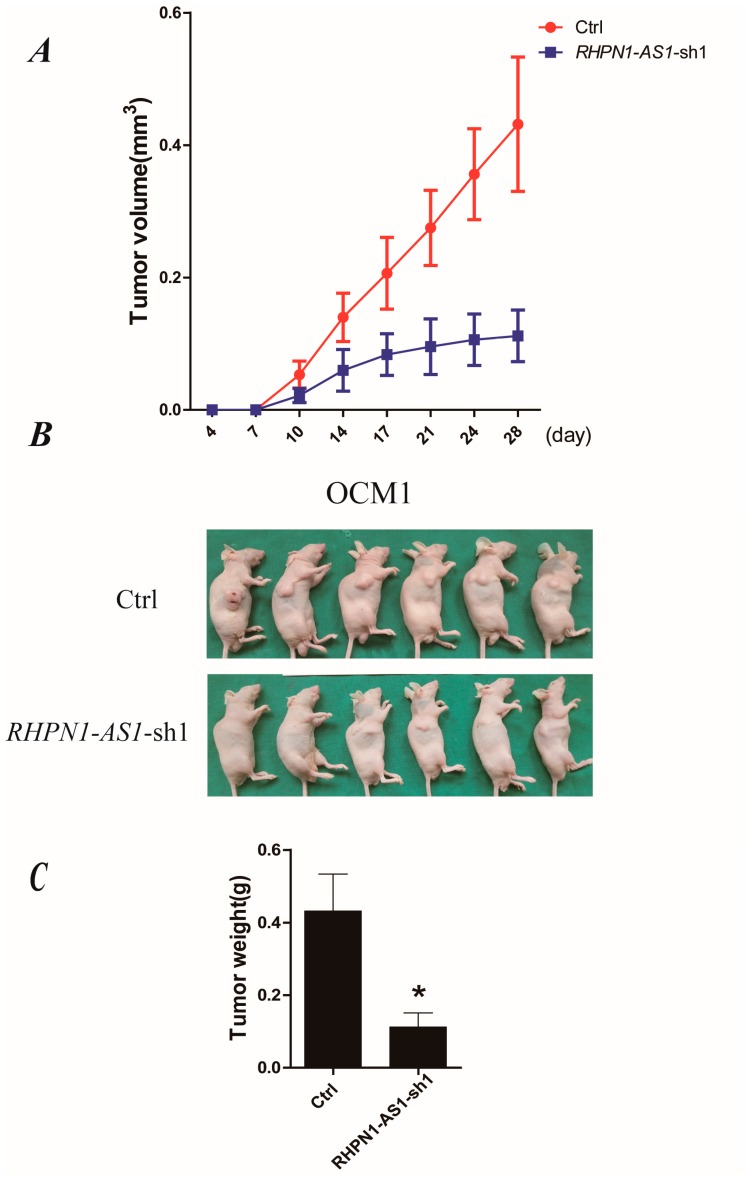
Depressed expression of *RHPN1-AS1* repressed UM cell growth in vivo. (**A**) Tumor volume growth curves. Each data point represents the mean ± SD; (**B**) *RHPN1-AS1* knockdown OCM1 and control cells were injected into nude mice subcutaneously. Representative images of tumor growth 28 days after subcutaneous injection; (**C**) Mean tumor weights four weeks after inoculation. * *p* < 0.05.

To examine the biological significance of *RHPN1-AS1* on tumor growth in vivo, we established a xenograft model in nude mice; 1 × 10^6^ of the control OCM1 or *RHPN1-AS1*-sh1OCM1 cells were subcutaneously injected into the right flanks of mice. The tumor volume was measured once every 3–4 days. Fourteen days after injection, the tumor growth of *RHPN1-AS1*-sh1OCM1 cells was significant slower than that of control cells (*p* < 0.05, Figure 4A). After 28 days, the mice were sacrificed, and the tumors were removed and analyzed (Figure 4B). The average tumor weight of *RHPN1-AS1-*sh1OCM1 cells was significantly lower than that of the control cells (*p* < 0.05, Figure 4C). These data indicate that the growth of the tumors was impaired after *RHPN1-AS1* knockdown in vivo.

### 2.5. Gene Expression Profile Analysis Revealed That Angiogenesis and Multiple Pathways May Be Downstream Targets Affected by RHPN1-AS1

**Figure 5:**
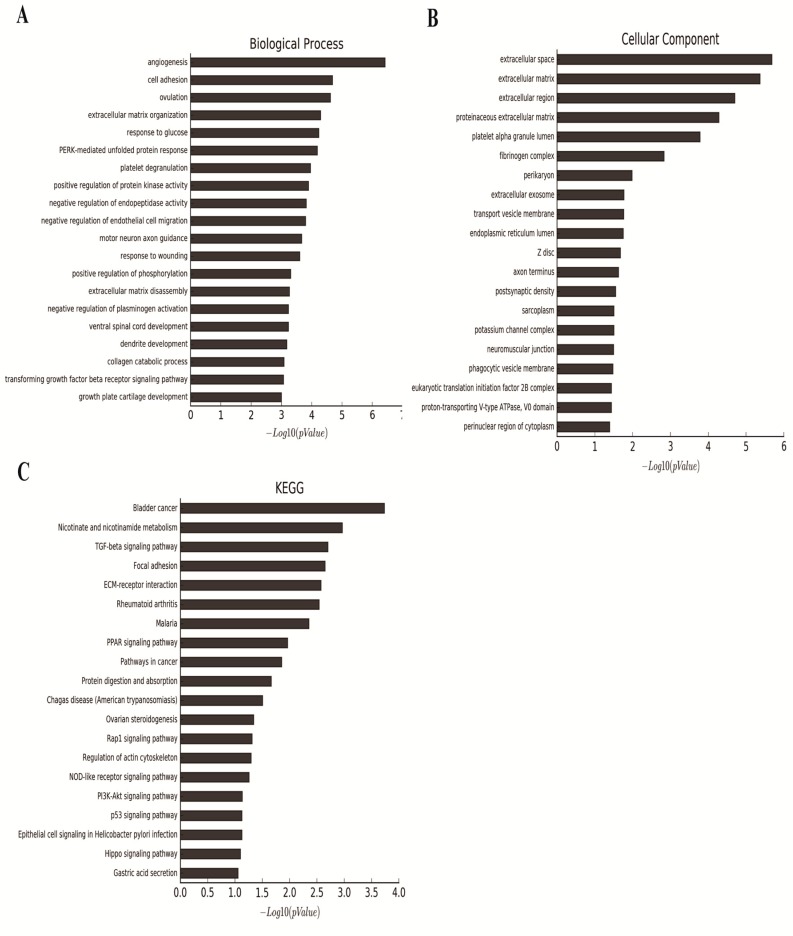
Genome-wide analysis of *RHPN1-AS1* targets. (**A**,**B**) Significant GOs affected by *RHPN1-AS1* silencing. *X*-axis, negative logarithm of the *p* value (−Lg*p*); *Y*-axis, the Gene Ontology (GO) category; (**C**) Histogram of signal pathways that were regulated by *RHPN1-AS1* silencing. *X*-axis, negative logarithm of the *p*-value (−Lg*p*); *Y*-axis, the name of the pathway.

To elucidate the mechanisms by which *RHPN1-AS1* contributes to the progression of UM, we carried out microarray analysis comparing the gene expression of OCM1 cells versus OCM1 cells stably transfected with *RHPN1-AS1-sh1*. Differentially expressed genes with at least a two-fold change were identified. The expression of 136 genes was altered compared with untreated tumor cells (Table S1). We functionally annotated these differentially expressed genes using gene ontology (GO). The categories involved angiogenesis, cell adhesion and extracellular matrix organization (Figure 5A,B). In addition, we identified significant pathways that mediated the functions of the differentially expressed genes based on the Kyoto Encyclopedia of Genes and Genomes (KEGG) database. The most significantly changed pathways included bladder cancer, nicotinate and nicotinamide metabolism, and TGF-β signaling pathway (Figure 5C). Notably, genes involved in TGF-β signaling pathway were significantly enriched, suggesting that *RHPN1-AS1* may participate in the TGF-β signaling pathway thus contributing to epithelial-to-mesenchymal transition (EMT). However, further studies are still needed to elucidate the mechanisms by which *RHPN1-AS1* modulates those processes and pathways.

## 3. Discussion

Melanoma is an aggressive malignant tumor of the melanocytes. Somatic mutations in the RAS/RAF/MEK/ERK signaling pathway are frequent in cutaneous melanomas, with 50%–70% of them being *BRAF* mutations [16]. However, *BRAF* mutations are rare in UM, and *GNAQ* and *GNA11* are typically mutated in UM. Thus, it is evident that aberrant signaling pathways in UM may be distinctive. Microarray assays and SNP assays have also confirmed the mutation frequencies of *BAP1*, *SF3B1* and *EIF1AX* in UM [17]. *GNAQ* is inversely associated with chromosome 3 monosomy and metastasis; and *BAP1* is significantly associated with chromosome 3 monosomy but not with relapse [17].

Global transcriptional analyses have revealed that the human genome is transcribed to produce a wide range of lncRNAs [18]. Recently, studies have highlighted that lncRNAs are connected with fundamental characteristics in a variety of tumors [11]. For example, prostate cancer associated transcript 1 (*PCAT-1*) is highly upregulated in metastatic prostate cancers. PCAT-1 knockdown increases cell proliferation, suggesting that *PCAT-1* might contribute to prostate cancer progression [19]. Through genome screening, Li et al. found that five ncRNA fragments interacted with the tumor suppressor PSF and released it from the proto-oncogene G antigen 6 (*GAGE6*) regulatory locus, resulting in an activation of *GAGE6* expression. Overexpression of these ncRNA fragments in melanoma cell lines led to enhanced tumorigenesis [20]. However, the role for lncRNAs in UM remains obscure. We compared the different expression profiles of lncRNAs between UM samples and normal tissues and identified a large number of aberrantly expressed lncRNAs in UM tissues (data not shown). *RHPN1-AS1* was highly expressed in UM tissues and cell lines, and was located at the 8q24.3, a region that is frequently amplified in a wide range of solid tumors, including UM. In this study, we found that the downregulation of *RHPN1-AS1* could significantly inhibit proliferation, migration and invasion of UM cells in vitro. Furthermore, the downregulation of *RHPN1-AS1* could inhibit tumor growth in murine model. Taken together, these findings indicated that *RHPN1-AS1* might serve as an oncoRNA and play an important role in UM progression.

It has been suggested that antisense RNAs could potentially regulate their corresponding sense mRNA at different level [21,22]. *RHPN1-AS1* is a ~2.03 kb RNA transcribed from the reverse strand of chromosome 8, on the opposite strand of the protein coding gene *RHPN1* (8q24.3), but there is no overlapping and complementary region between *RHPN1-AS1* and *RHPN1*. To determine the relationship between *RHPN1-AS1* and *RHPN1*, we examined the mRNA and protein level of *RHPN1* after knocking down endogenous *RHPN1-AS1* expression in OCM1 and OM431 cells using qPCR and western blot. The results indicated that down-regulated expression of *RHPN1-AS1* did not significantly affect *RHPN1* expression (Figure S2), suggesting that the biological effect of *RHPN1-AS1* is independent of *RHPN1*.

Previous studies have shown that not only can lncRNAs govern expression of neighboring protein-coding genes (*cis-*acting regulation), but also they can regulate distal transcriptional elements (*trans-*acting) through various mechanisms [23]. Given that expression of *RHPN1* did not change after *RHPN1-AS1* knockdown, we further explored the genes and pathways that *RHPN1-AS1* may regulate using microarray analysis. GO analysis revealed that differentially expressed genes after *RHPN1-AS1* knockdown are associated with biological processes including angiogenesis, cell adhesion and extracellular matrix organization. Pathway analysis using the KEGG database revealed that the most significantly changed pathways included bladder cancer, nicotinate and nicotinamide metabolism and TGF-β signaling pathway. It is noteworthy that TGF-β signaling pathway, which has a tremendous impact on the regulation of EMT is significantly changed after *RHPN1-AS1* knockdown. Evidence suggests that epithelial-to-mesenchymal transition (EMT) participates during the progression of cancer, and TGF-β signaling promotes EMT by initiating the process and establishing a dramatic cellular adaptation that permeates a great number of vital cell biological processes [24]. Therefore, we hypothesize that RHPN1-AS1 may promote UM progression at least partly through interplay with TGF-β signaling pathway. However, the specific cellular processes that are directly affected by alterations in *RHPN1-AS1* expression remain to be elucidated.

Accumulating studies have shown that lncRNAs act as crucial regulators in tumor progression [25,26]. Therefore, lncRNAs could be one of the leading forces during UM tumorigenesis. Identification of UM-associated lncRNAs and investigation of their biological functions and clinical significance may provide a strategy and facilitate the development of lncRNA-directed diagnosis and improved prognosis of UM.

## 4. Materials and Methods

### 4.1. Cell Culture and Short Interfering RNAs (siRNAs) in UM Cells

OM431, OCM1 and RPE cells were cultured in Dulbecco’s Modified Essential Medium (DMEM; Gibco, Carlsbad, CA, USA) with 10% fetal bovine serum (FBS) and maintained at 37 °C in a 5% CO_2_ atmosphere. Three different siRNAs targeting *RHPN1-AS1* and a scrambled siRNA mock were designed and synthesized (Biotend, Shanghai, China). In total, 2 × 10^5^ UM cells were seeded into six-well plates and transfected with the siRNAs using Lipofectamine 2000 (Invitrogen, Carlsbad, CA, USA) according to the manufacturer’s protocol. The nucleotide sequences of the siRNAs for *RHPN1-AS1* are shown in Table 1. Transfection efficiency was optimized using fluorescein labeled negative controls. Cells were harvested at 48 h.

### 4.2. RNA Extraction and Quantitative Real-Time Polymerase Chain Reaction (qRT-PCR) Assays

Total RNA was isolated using Trizol reagent (Invitrogen) following the manufacturer’s protocol. For qRT-PCR, RNA was reverse transcribed to cDNA using a Reverse Transcription Kit (Takara, Dalian, China). Screening for lncRNAs was performed by qRT-PCR with the primer sets described in Table 1. Real-time PCR analyses were performed using SYBR Premix Ex Taq (Takara), which was repeated at least three times for each sample. Gene expression levels were normalized to that of *GAPDH*. The qRT-PCR assays were conducted on a Roche PCR instrument.

### 4.3. FluorescenceIn Situ Hybridization

*RHPN1-AS1*-Fish Probe Mix was purchased from RiboBio Corporation (Guangzhou, China), and Fluorescence in situ hybridization was performed using Fluorescent In Situ Hybridization Kit (RiboBio Co., Guangzhou, China). Briefly, OCM1 cells grown on cover slips were fixed with 4% formaldehyde for 10 min, and then permeabilized with 0.5% Triton X-100 in phosphate-buffered saline (PBS) for 20 min. For hybridization, the cells were incubated in hybridization mixtures at 37 °C for 30 min before probes were added. After incubation with *RHPN1-AS1*-Fish Probe Mix at 37 °C overnight, the cells were washed with 4× saline sodium citrate (SSC; containing 0.1% Tween-20) at 42 °C for 5 min, followed by 2× SSC at 42 °C for 5min and 1× SSC at 42 °C for 5 min. The nuclei were counterstained with 4′,6-diamidino-2-phenylindole (DAPI), after which images were taken.

### 4.4. Short Hairpin (sh) RNA-Expressing Plasmid Construction, Lentivirus Packaging, Cloning and Stable Transfection

To reduce the expression of *RHPN1-AS1*, two human *RHPN1-AS1* shRNA sequences 5′-CCGAATCTCTTTACTTCCA-3′ and 5′-CTCAAACTTTGAGGGTCAT-3′ were cloned into the pGIPZ-lentivirus vector (System Biosciences, Palo Alto, CA, USA). Thereafter, two *RHPN1-AS1* knockdown vectors, namely p*GIPZ-RHPN1-AS1-sh1* and p*GIPZ-RHPN1-AS1-sh2*, were constructed and sequenced (Figure S3). The empty pGIPZ vector without any insertion was used as a control. 293T cells were cultured in DMEM containing 10% FBS, maintained at 37 °C and transfected using Lipofectamine 2000 reagent (Invitrogen, Carlsbad, CA, USA) with 3 µg p*GIPZ-RHPN1-AS1-sh1* or *-sh2*, 6.0 µg PsPax2 and 3 µg pMD 2.G. The media were replaced with 10 mL fresh medium after incubation overnight. The virus-containing supernatants were collected at 48 and 72 h. OCM1 and OM431 cells were infected and then selected using 4 μg/mL puromycin. The knockdown efficiency was measured by qRT-PCR.

### 4.5. Cell Migration Assay

Totally, 1 × 10^5^ cells in serum-free media were placed into the upper chamber of each insert (8.0 µm, Millipore, Palo Alto, MA, USA). Then the chambers were incubated for 36 h in 600 μL culture medium supplemented with 10% FBS in the bottom chambers before examination. The cells on the upper surface were scraped off with a cotton swab, and the migrated cells on the lower surface were fixed 4% paraformaldehyde and stained with 5% crystal violet. After washing with PBS, images were taken. Experiments were independently repeated in triplicate.

### 4.6. Cell Invasion Assay

Matrigel-coated chambers (BD Biosciences, Palo Alto, CA, USA) with 8 μm pores were used for invasion assay. Two hundred microliters cells in serum-free medium were seeded in the upper chambers at 5 × 10^4^/mL concentration. The lower chamber was filled with 800 μL DMEM medium containing 10% FBS. After incubation at 37 °C for 24 h, non-invaded cells on the top of the wells were scraped off, whereas the translocated cells on the lower surface were fixed with 4% paraformaldehyde and stained with 5% crystal violet. After washing with PBS, cells were counted under a light microscope (Olympus, Tokyo, Japan).

### 4.7. Colony Formation Assay

In total, 2 × 10^3^ cells were placed in each well of the six-well plates and maintained in DMEM containing 10% FBS at 37 °C for 8 days. After 8 days, the cells were fixed with 4% paraformaldehyde and stained with 5% crystal violet. Visible colonies were then counted and imaged. Experiments were independently repeated three times.

### 4.8. Tumor Xenograft Model in Nude Mice

OCM1 cells stably expressing *RHPN1-AS1-sh1* and control OCM1 cells (1 × 10^6^) were subcutaneously injected at 100 µL cell suspension per injection into the right flanks of 3-week-old athymic nude mice (*n* = 6 per group). The mice were housed under a controlled environment in a sterile facility. The tumor volume was measured once every 3–4 days with calipers. Tumor volume was calculated using the following formula: 0.5 × length × width × height. After 28 days, the mice were sacrificed, and the tumors were removed and analyzed. The Animal Care and Use Committee at Ninth People’ Hospital affiliated to Shanghai Jiao Tong University, School of Medicine approved the animal protocols (HKDL[2014]70, 25 February 2014).

### 4.9. Nuclear/Cytoplasmic RNA Fractionation

OCM1 cells were grown in 10 cm dishes. After reaching 80% confluence, cells were centrifuged at 800 rpm for 5 min and rinsed with ice-cold PBS. Nuclear/cytoplasmic RNA fractionation was performed using Nuclear/Cytosol Fractionation Kit (BioVision Inc., Milpitas, CA, USA) according to manufacturer’s protocol. After cytoplasmic fraction and nuclear fraction were isolated, RNAs were extracted by Trizol reagent (Invitrogen). Semi-quantitative RT-PCR was then performed to evaluate the relative abundance of *RHPN1-AS1*, *U1* and *GAPDH* in each sample.

### 4.10. DNA Microarray Analysis and Data Analysis

The AffymetrixPrimeView™ Human Gene Expression Array (Affymetrix, Santa Clara, CA, USA) was used in this experiment. Total RNA of OCM1 cells and OCM1 cells stably transfected with *RHPN1-AS1-sh1* was isolated using the TRIzol reagent (Invitrogen, Carlsbad, CA, USA). Total RNA was quantified using NanoDrop ND-2000 (Thermo Scientific, Waltham, MA, USA) and the RNA integrity was assessed by the Agilent Bioanalyzer 2100 (Agilent Technologies, Santa Clara, CA, USA). The sample labeling, microarray hybridization and washing were performed according to the manufacturer’s standard protocols. Briefly, total RNA were transcribed to double strand cDNA, then synthesized cRNA and labeled with biotin. The labeled cRNAs were hybridized onto the microarray. After washing and staining, the arrays were scanned by the Affymetrix Scanner 3000 (Affymetrix, Santa Clara, CA, USA). Affymetrix GeneChip Command Console (version 4.0, Affymetrix) was employed to analyze array images to get raw data, which was then analyzed using Genesping software (version 13.1; Agilent Technologies). First, the raw data was normalized with the RMA algorithm. Through fold change and p value calculated by *t*-test, differentially expressed genes were identified. A threshold of fold change ≥2.0 and *p*-value ≤ 0.05 was set for identifying up- and down-regulated genes. Afterwards, Gene Ontology (GO) database and Kyoto Encyclopedia of Genes and Genomes (KEGG) database were used for the functional annotation and enrichment analysis of these differentially expressed mRNAs.

### 4.11. Statistical Analysis

All statistical analyses were performed using SPSS version 19.0 software (SPSS, Inc., Chicago, IL, USA). *p*-values < 0.05 were considered to be statistically significant.

## 5. Conclusions

In this study, we verified that *RHPN1-AS1* was upregulated in UM cancerous tissues and plays a vital role in UM cell proliferation, migration and invasion. Depletion of *RHPN1-AS1* inhibited the growth of UM in vitro and in vivo. Microarray analysis further identified multiple pathways and biological processes that might be responsible for UM tumor growth and metastasis that were altered by *RHPN1-AS1*. These results suggest *RHPN1-AS1* may be an attractive biomarker and therapeutic target in UM.

## Figures and Tables

**Table 1:** Primers and siRNA used in this study.

Primer Name	Sequence (5′–3′)	Purpose
*RHPN1-AS1*-F	GCTCCTGGTCATCAAGTTCCTCT	qRT-PCR
*RHPN1-AS1*-R	GCACAGGCACCAGAATGATCC	qRT-PCR
*RHPN1*-F	TACGACTCGCTTACTGGGGT	qRT-PCR
*RHPN1*-R	GAGGGCACCGATGTTGAAGA	qRT-PCR
*GAPDH*-F	AGGTCGGAGTCAACGGATTTG	qRT-PCR
*GAPDH*-R	TGTAAACCATGTAGTTGAGGTCA	qRT-PCR
*RHPN1-AS1-*si1-sense	CCGAAUCUCUUUACUUCCAdTdT	si*RHPN1-AS1*
*RHPN1-AS1*-si1-antisense	UGGAAGUAAAGAGAUUCGGdTdT	si*RHPN1-AS1*
*RHPN1-AS1*-si2-sense	CUCAAACUUUGAGGGUCAUdTdT	si*RHPN1-AS1*
*RHPN1-AS1*-si2-antisense	AUGACCCUCAAAGUUUGAGdTdT	si*RHPN1-AS1*
*RHPN1-AS1*-si3-sense	CUUCCAUACUUCCCUAGGUdTdT	si*RHPN1-AS1*
*RHPN1-AS1*-si3-antisense	ACCUAGGGAAGUAUGGAAGdTdT	si*RHPN1-AS1*

## Supplementary Materials

Supplementary materials can be found at www.mdpi.com/1422-0067/18/1/226/s1.
