# Supplementary material for: The Long Non-Coding RNA RHPN1-AS1 Promotes Uveal Melanoma Progression
Source: Int J Mol Sci. 2017 Jan 23;18(1):226. doi: 10.3390/ijms18010226 (PMC5297855; doi:10.3390/ijms18010226)
Supplement: Supplementary file 1 [file ijms-18-00226-s001.pdf]

Supplementary Materials: The Long Non-Coding RNA *RHPN1-AS1* Promotes Uveal Melanoma Progression

Linna Lu, Xiaoyu Yu, Leilei Zhang, Xia Ding, Hui Pan, Xuyang Wen, Shiqiong Xu, Yue Xing, Jiayan Fan, Shengfang Ge, He Zhang, Renbing Jia and Xianqun Fan

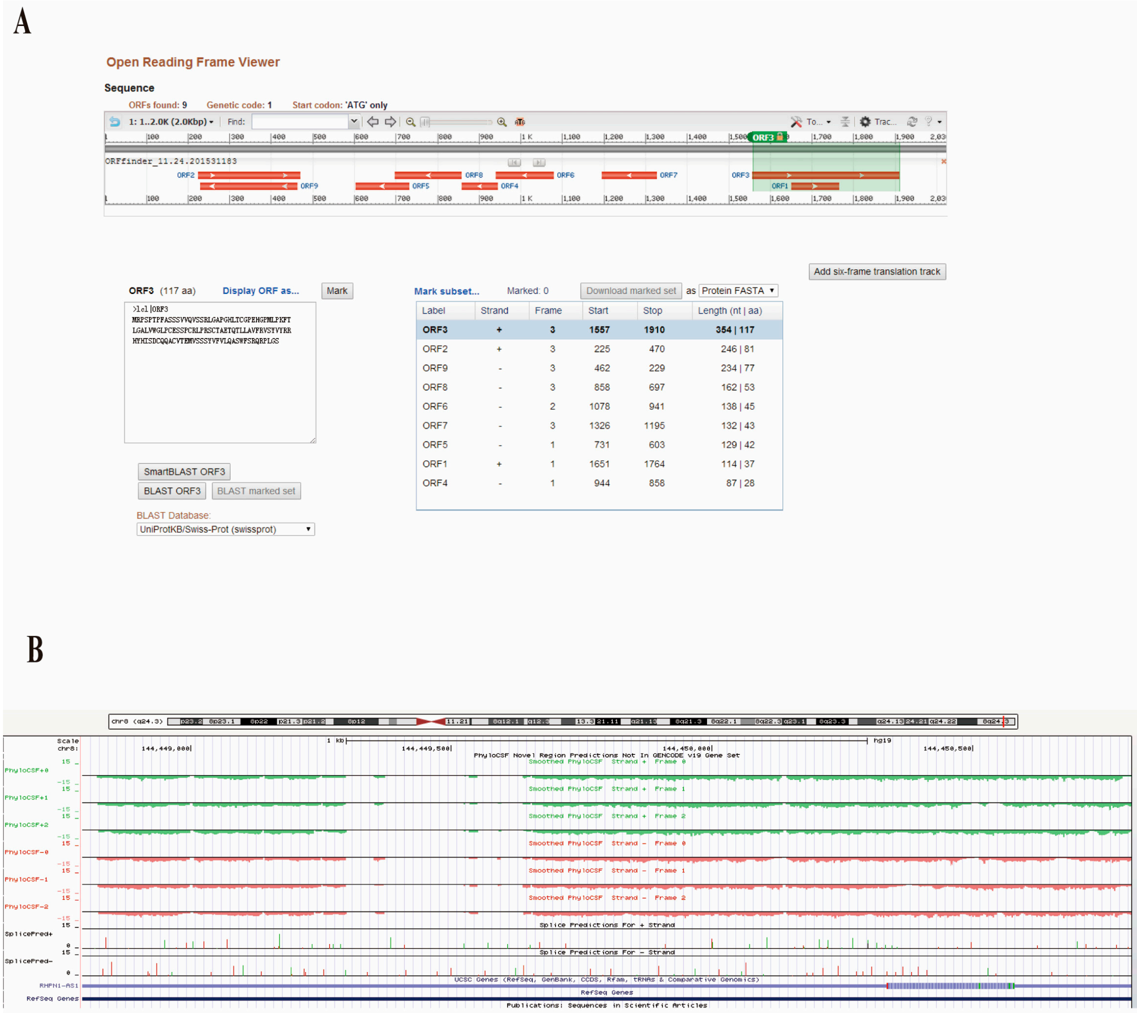

C

Data ID	Sequence Name	RNA Size	ORF Size	Ficket Score	Hexamer Score	Coding Probability	Coding Label
0	NR_026785.1	2030	354	0.4988	-0.136101002674	0.023563496365207	no

Figure S1. Coding-potential analysis of *RHPN1-AS1*: (**A**) prediction of putative proteins encoded by lncRNA*RHPN1-AS1* using Open Reading Frame Finder; (**B**) the condon substitution frequency scores (CSF) of lncRNA*RHPN1-AS1*; and (**C**) protein coding potentials predicted by Coding Potential Assessment Tool (CPAT) program (Available online: http://lilab.research.bcm.edu/cpat/).

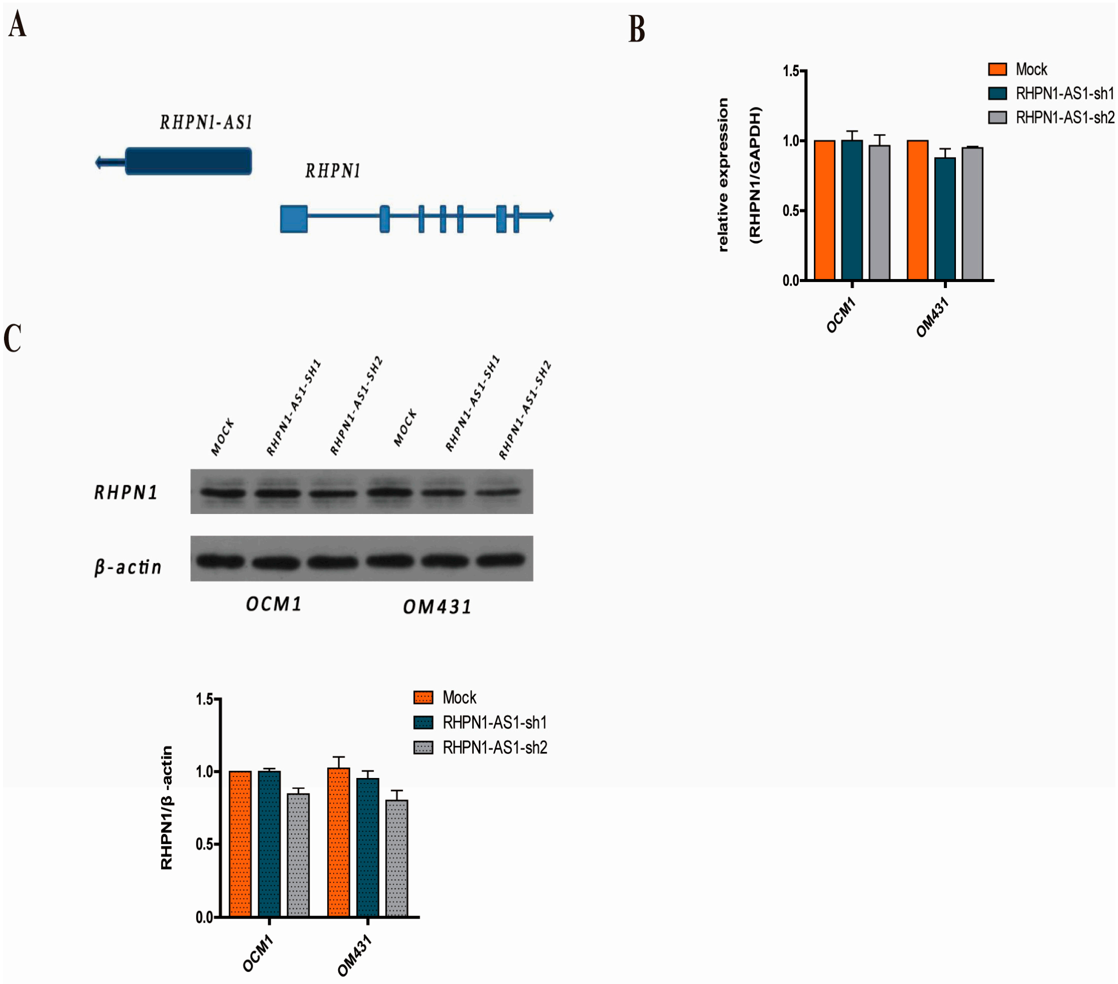

Figure S2. The expression of *RHPN1* after *RHPN1-AS1* knockdown: (**A**) Schematic illustration of *RHPN1-AS1* and *RHPN1*. There is no overlapping and complementary region between *RHPN1-AS1* and *RHPN1*; (**B**,**C**) The mRNA and protein levels of *RHPN1* after *RHPN1-AS1* knockdown in UM cell lines examined by: real-time RT-PCR (**B**); and Western blot (**C**).

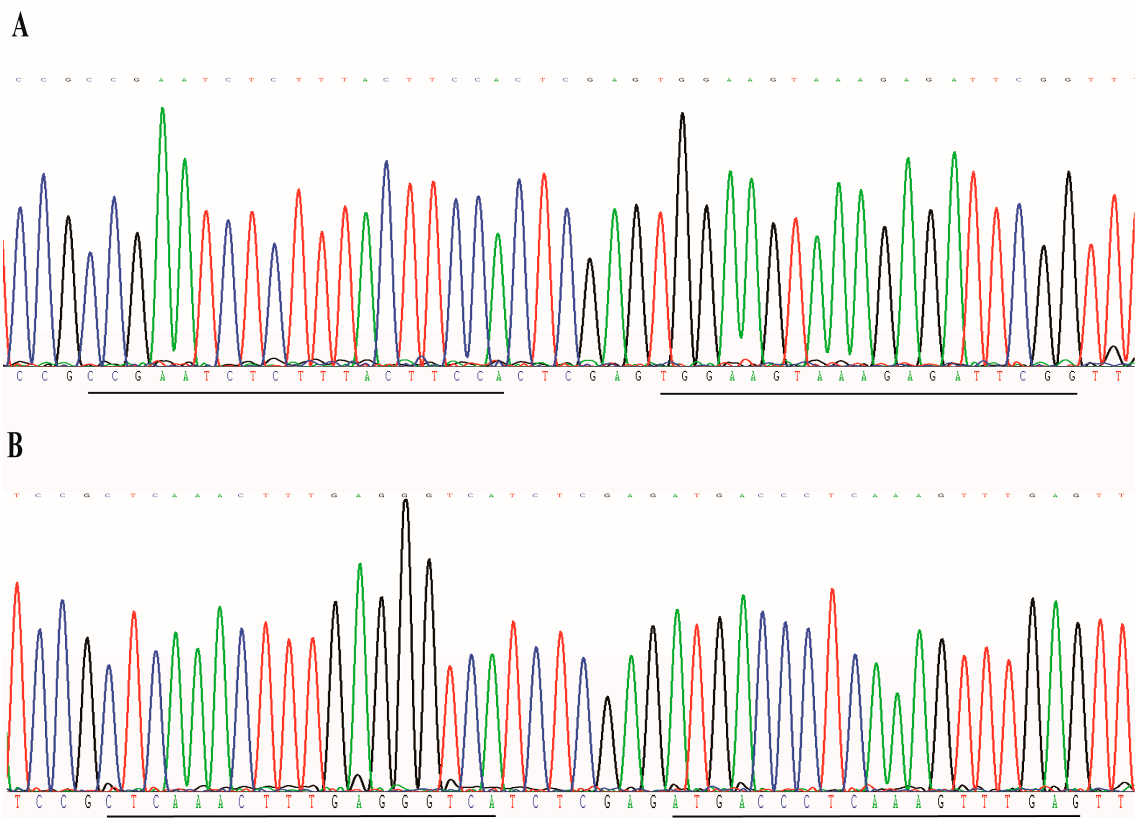

Figure S3. Sequencing of the GIPZ-*shRHPN1-AS1* plasmids. Black lines denoted the shRNA sequence of: *RHPN1-AS1-sh1* (**A**); and *RHPN1-AS1-sh2* (**B**).

Table S1. Altered genes in the cDNA microarray after *RHPN1-AS1* knockdown.

Ensembl Gene ID	Gene Symbol	Fold Change (abs)
ENSG00000196611	*MMP1*	14.781309
ENSG00000196611	*MMP1*	11.014036
ENSG00000175445	*LPL*	8.477454
ENSG00000175445	*LPL*	6.9571214
ENSG00000108691	*CCL2*	6.3504324
ENSG00000205097	*FRG2*	4.5775504
ENSG00000099250	*NRP1*	3.762244
ENSG00000159167	*STC1*	3.6682963
ENSG00000172969	*FLJ20518*	3.619705
ENSG00000163359	*COL6A3*	3.4515817
ENSG00000144837	*PLA1A*	3.28566
ENSG00000147614	*ATP6V0D2*	3.2046456
ENSG00000175445	*LPL*	3.1958368
ENSG00000019505	*SYT13*	3.155073
ENSG00000227304	*RPS6P6*	3.141476
ENSG00000166741	*NNMT*	3.1386187
ENSG00000166750	*SLFN5*	3.043957
ENSG00000134775	*FHOD3*	3.0215907
ENSG00000148828	*FRG2*	3.019656
ENSG00000185985	*SLITRK2*	2.9938574
ENSG00000178150	*ZNF114*	2.9920273
ENSG00000138798	*EGF*	2.9560235

Table S1. *Cont.*

Ensembl Gene ID	Gene Symbol	Fold Change (abs)
ENSG00000182583	*VCX*	2.93854
ENSG00000106366	*SERPINE1*	2.9334047
ENSG00000118523	*CTGF*	2.923989
ENSG00000198157	*HMGN5*	2.8985822
ENSG00000169059	*VCX*	2.8471634
ENSG00000146250	*PRSS35*	2.8438642
ENSG00000109472	*CPE*	2.8314576
ENSG00000112280	*COL9A1*	2.7647889
ENSG00000117228	*GBP1*	2.7140744
ENSG00000111341	*MGP*	2.6919894
ENSG00000137801	*THBS1*	2.6883326
ENSG00000104112	*SCG3*	2.6847403
ENSG00000196139	*AKR1C3*	2.6694527
ENSG00000170571	*EMB*	2.6405358
ENSG00000186462	*NAP1L2*	2.6251915
ENSG00000118523	*CTGF*	2.6143558
ENSG00000168824	*D4S234E*	2.529696
ENSG00000120708	*LOC100652886*	2.5225365
ENSG00000118523	*CTGF*	2.5169957
ENSG00000215417	*MIR17HG*	2.512744
ENSG00000169282	*KCNAB1*	2.5121071
ENSG00000120738	*EGR1*	2.497552
ENSG00000197594	*ENPP1*	2.4634533
ENSG00000153253	*SCN3A*	2.42825
ENSG00000178150	*ZNF114*	2.4245365
ENSG00000156675	*RAB11FIP1*	2.4116204
ENSG00000169429	*IL8*	2.4049144
ENSG00000079385	*CEACAM1*	2.3992805
ENSG00000115414	*FN1*	2.3826158
ENSG00000145703	*IQGAP2*	2.3777382
ENSG00000116106	*EPHA4*	2.3715253
ENSG00000170017	*ALCAM*	2.364987
ENSG00000184564	*SLITRK6*	2.3384037
ENSG00000115414	*FN1*	2.317143
ENSG00000057704	*TMCC3*	2.308804
ENSG00000170571	*EMB*	2.3037548
ENSG00000169282	*KCNAB1*	2.3001454
ENSG00000184903	*IMMP2L*	2.2926645
ENSG00000188419	*CHM*	2.2834475
ENSG00000105519	*CAPS*	2.2812297
ENSG00000115414	*FN1*	2.2771678
ENSG00000117318	*ID3*	2.2768095
ENSG00000141469	*SLC14A1*	2.2700348
ENSG00000164627	*KIF6*	2.2642193
ENSG00000164953	*TMEM67*	2.26346
ENSG00000078295	*ADCY2*	2.2551606
ENSG00000197467	*COL13A1*	2.2550316
ENSG00000169551	*CXorf48*	2.2550309

Table S1. *Cont.*

Ensembl Gene ID	Gene Symbol	Fold Change (abs)
ENSG00000122367	*LDB3*	2.2353542
ENSG00000137834	*SMAD6*	2.2292302
ENSG00000115414	*FN1*	2.2275038
ENSG00000065534	*MYLK*	2.2249124
ENSG00000138347	*MYPN*	2.2223063
ENSG00000122877	*EGR2*	2.221315
ENSG00000124882	*EREG*	2.220559
ENSG00000168843	*FSTL5*	2.2182937
ENSG00000254685	*FPGT*	2.2180545
ENSG00000174374	*LOC100505669*	2.2095828
ENSG00000196154	*S100A4*	2.2038631
ENSG00000111361	*EIF2B1*	2.2036345
ENSG00000163359	*COL6A3*	2.1855834
ENSG00000139800	*ZIC5*	2.1824057
ENSG00000164120	*HPGD*	2.1770177
ENSG00000152377	*SPOCK1*	2.175735
ENSG00000154274	*C4orf19*	2.1680813
ENSG00000107984	*DKK1*	2.166239
ENSG00000050344	*NFE2L3*	2.1657064
ENSG00000055957	*ITIH1*	2.158894
ENSG00000138798	*EGF*	2.1579823
ENSG00000196950	*SLC39A10*	2.1494274
ENSG00000079385	*CEACAM1*	2.1488094
ENSG00000205413	*SAMD9*	2.1395016
ENSG00000134160	*TRPM1*	2.1351733
ENSG00000147614	*ATP6V0D2*	2.132258
ENSG00000125968	*ID1*	2.1321712
ENSG00000170017	*ALCAM*	2.1318789
ENSG00000141469	*SLC14A1*	2.1265373
ENSG00000174928	*C3orf33*	2.119451
ENSG00000147027	*TMEM47*	2.1180506
ENSG00000115896	*PLCL1*	2.1140459
ENSG00000159167	*STC1*	2.1138396
ENSG00000164120	*HPGD*	2.1137419
ENSG00000113396	*SLC27A6*	2.104763
ENSG00000151632	*AKR1C1*	2.1029902
ENSG00000085276	*MECOM*	2.1010337
ENSG00000124831	*LRRFIP1*	2.0972662
ENSG00000115414	*FN1*	2.0936246
ENSG00000070526	*ST6GALNAC1*	2.0915484
ENSG00000117650	*NEK2*	2.089459
ENSG00000115414	*FN1*	2.0888126
ENSG00000160862	*AZGP1*	2.0806043
ENSG00000115414	*FN1*	2.0684946
ENSG00000070669	*ASNS*	2.0653944
ENSG00000189056	*RELN*	2.0653806
ENSG00000000971	*CFH*	2.063501
ENSG00000147041	*SYTL5*	2.0634027

Table S1. *Cont.*

Ensembl Gene ID	Gene Symbol	Fold Change (abs)
ENSG00000122707	*RECK*	2.0590112
ENSG00000176853	*FAM91A1*	2.0588403
ENSG00000171368	*TPPP*	2.0540159
ENSG00000196154	*S100A4*	2.049158
ENSG00000164120	*HPGD*	2.0442598
ENSG00000171522	*PTGER4*	2.036862
ENSG00000132386	*SERPINF1*	2.0365548
ENSG00000253767	*PCDHGA8*	2.0360367
ENSG00000089048	*ESF1*	2.0290923
ENSG00000183508	*FAM46C*	2.0276382
ENSG00000100426	*ZBED4*	2.025417
ENSG00000116922	*C1orf109*	2.0216181
ENSG00000173406	*DAB1*	2.021513
ENSG00000142871	*CYR61*	2.0197327
ENSG00000134955	*SLC37A2*	2.0127854
ENSG00000070669	*ASNS*	2.0124888
ENSG00000065534	*MYLK*	2.0036278
ENSG00000112874	*NUDT12*	2.0024552
